# Sensorless Model Predictive Control of Single-Phase Inverter for UPS Applications via Accurate Load Current Estimation

**DOI:** 10.3390/s23073742

**Published:** 2023-04-04

**Authors:** Po Li, Xiaoshan Tong, Zhoujing Wang, Maoguang Xu, Jianfeng Zhu

**Affiliations:** School of Aerospace Engineering, Xiamen University, Xiamen 361102, China

**Keywords:** load current estimation, observer, model predictive control, uninterruptible power supply, load current sensorless control

## Abstract

Single-phase inverters with an output LC filter, can generate low distortion output voltages, which are suitable for uninterruptible power supply (UPS) systems. The UPS system provides emergency power in the case of utility power failure, requiring high reliability and clean power. The sensorless control method is actually a soft-sensing technique, that reduces system cost, measurement-related losses, and, especially important for UPS systems, enhances the system reliability. This paper proposes a load current sensorless finite control set model predictive control (FCS-MPC) scheme for a single-phase UPS inverter. A time varying observer is proposed, which offers the accurate estimation for individual components simultaneously in periodic load current signal, without subsequent complex calculations. Compared with another two typical sensorless methods (the low-pass filter and the Kalman filter), the proposed observer-based FCS-MPC strategy has smaller load current estimation error and lower output voltage distortion, under both linear and nonlinear loads. The theoretical analysis is verified through simulation and experiment. A single-phase inverter rapid control prototype (RCP) is set up with the Speedgoat real-time target machine, to confirm the effectiveness of the system.

## 1. Introduction

The control of a single-phase inverter is a common topic in power electronics and has been extensively studied [1,2]. The use of an inverter with an output LC filter brings a low distortion output voltage, which is very suitable for occasions that require high output voltage quality, such as uninterruptible power supply (UPS) systems. UPS provides emergency power to loads when the utility power system fails. UPS systems are becoming more and more important, due to the increase in sensitive and critical applications such as communication systems, medical equipment, semiconductor manufacturing systems, and data processing systems [3,4,5]. High reliability and clean power are crucial for these applications.

Many control methods have been proposed for inverters [6,7], including linear methods, such as proportional integral (PI) and proportional resonant (PR) controllers [8,9,10], and nonlinear methods, such as sliding mode control [11], direct power control, and hysteresis current control [12]. Model predictive control (MPC) methods have become some of the most well-known methods [13,14,15]. Among them, the finite control set model predictive control (FCS-MPC), gives full play to the finite switching state characteristic of power electronic converters, and has the advantages of no modulation unit, no need to consider the prediction, and a control time domain [16,17]. Therefore, it has been widely studied and applied [18], particularly for inverters with an LC filter [19,20,21]. In an effort to obtain better steady and dynamic performances and lower THD, many studies have been performed [22,23]. For example, Ref. [24] presents the effect of considering different numbers of prediction steps in terms of THD and the number of cycles. However, the longer prediction horizons increase the computational cost significantly. To solve this problem, an improved FCS-MPC strategy, using only a one-step prediction horizon, is introduced in [25]. Besides, for single-phase inverters with an LC filter, a two-step prediction FCS-MPC algorithm is presented in [26] for stand-alone renewable power systems.

Accurate control of a typical FCS-MPC scheme for UPS inverters with an LC filter, requires sensing of the inductor and load currents, and output voltage. UPS systems require high reliability, while physical sensors are easily subject to noise, phase lag, and have a limited lifespan, which reduces the control precision and the system reliability [27]. A sensorless control method, is actually a soft-sensing technique, that reduces measurement-related losses and system cost. In addition, it works as a back up plan in case of sensor faults, guaranteeing the system’s continuous operation, which is especially important for UPS systems.

Because of these advantages, sensorless control has been extensively studied, to reduce the number of sensors in inverters. The Kalman filter estimator, which is a common estimation method, was used to estimate the instantaneous grid voltage in [28,29]. They designed a Kalman filter gain to achieve stability and eliminate system noise, but this brings complex calculations. As for observer methods, observer-based load current sensorless control was introduced for inverters with an LC filter, to reduce the system cost in [19,22,30]. In [31], an observer and a simple control strategy were proposed, to estimate the capacitor voltage and reduce the control complexity for a three-phase FCI. Furthermore, when the sensorless control is combined with the FCS-MPC method, the structure is more compact and the reliability of the system becomes higher, compared with a conventional linear control strategy with a modulator [32]. For voltage sensorless methods, a sliding-mode grid voltage observer was proposed in [33], and this turned out to be a compact solution for LC-filtered voltage source converters without voltage sensors. As for current sensorless methods, an observer-based current sensorless FCS-MPC scheme was designed for three-phase voltage source inverters with LC filters in [27]. While for single-phase inverters, due to the lack of corresponding coordinate transformation, the single-phase sensorless method is actually much more difficult. In [34], an observer-based capacitor current sensorless control was adopted in a seamless transfer single-phase inverter, but the observer equation was complex leading to relatively complex calculations.

In this paper, an enhanced sensorless control scheme of single-phase with an LC filter, for UPS applications, via accurate load current estimation, is proposed. Firstly, based on the mathematical model, a linear convergent time varying observer is proposed, to estimate the load current. It can quickly detect individual harmonic components in load current simultaneously, without subsequent complex calculations. Secondly, the two-step model predictive control system is introduced. Thirdly, comparisons among the proposed observer-based load current sensorless FCS-MPC strategy and another two sensorless FCS-MPC methods: the inverse calculation method cascaded the low-pass filter and the Kalman filter, are made through simulations and experiments, with both liner and nonlinear loads. A single-phase inverter rapid control prototype (RCP) is set up with the Speedgoat real-time target machine, to validate the proposed system.

## 2. System Description

### 2.1. System Model

**Figure 1:**
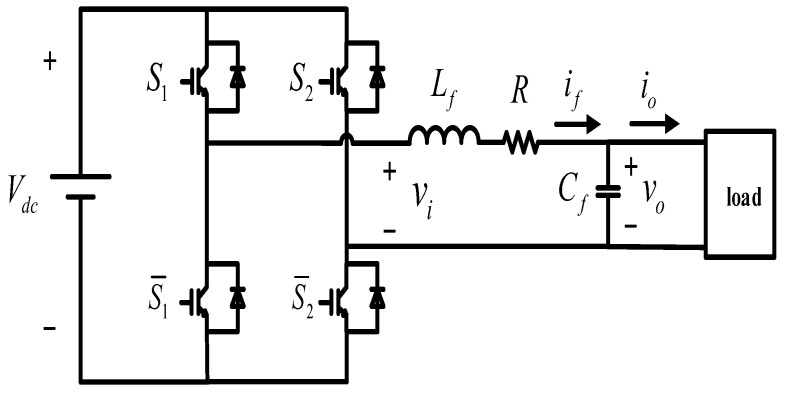
Single-phase inverter with output LC filter [35].

**Figure 2:**
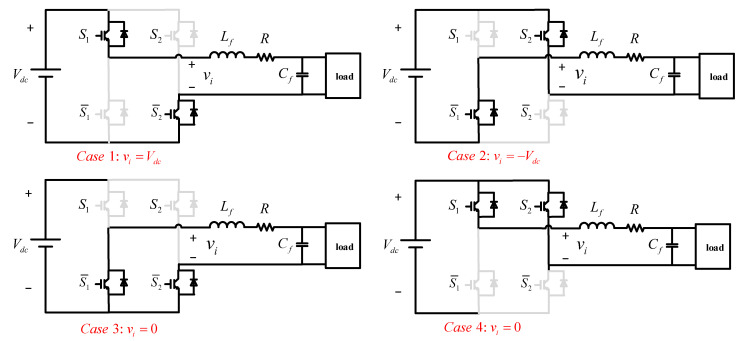
Operation modes of single-phase inverter with output LC filter.

The studied single-phase inverter with LC filter is presented in Figure 1. The left side of the single-phase H-bridge inverter is a DC voltage source, and the right side is the unknown load, connected through an LC filter. $V_{dc}$ and $S_{1},S_{2},{\bar{S}}_{1},{\bar{S}}_{2}$ represent the DC voltage source and four switches. The states of $S_{1}$ and $S_{2}$ are the opposite of ${\bar{S}}_{1}$ and ${\bar{S}}_{2}$, respectively. There are four operation modes of circuit in total, which are shown in Figure 2.

The H-bridge output voltages $v_{i}$ are $V_{dc}$, $-V_{dc}$, 0, and 0, for Case 1–4, respectively. Then, it can be derived as follows:

(1) $$\begin{array}{c} v_{i}=\left\{\begin{array}{cc} V_{dc} & S_{1}=1,S_{2}=0,\\ 0 & S_{1}=0,\;S_{2}=0\;\mathrm{or}\;S_{1}=1,S_{2}=1,\\ -V_{dc} & S_{1}=0,S_{2}=1, \end{array}\right. \end{array}$$

### 2.2. LC Filter Model

$C_{f}$ and $L_{f}$ form an LC filter, whose main function is to filter out higher harmonics in the system current. The filter differential equation is shown in (2) and (3):

(2) $$v_{o}=v_{i}-L_{f}\frac{di_{f}}{dt}-Ri_{f}$$

(3) $$i_{o}=i_{f}-C_{f}\frac{dv_{o}}{dt}$$

where *R* is the internal resistance of the system, $i_{f}$ is the filter input current, $i_{o}$ is the load current, and $v_{o}$ is the output voltage. Based on the one-step Euler equation with the sampling time $T_{s}$, (2) and (3) can be rewritten as (4) and (5), respectively.

(4) $$v_{o}\left(k\right)=v_{i}\left(k\right)-L_{f}\frac{i_{f}\left(k+1\right)-i_{f}\left(k\right)}{T_{s}}-Ri_{f}\left(k\right),$$

(5) $$i_{o}\left(k\right)=i_{f}\left(k\right)-C_{f}\frac{v_{o}\left(k+1\right)-v_{o}\left(k\right)}{T_{s}}.$$

## 3. Two-Step Model Predictive Control

**Figure 3:**
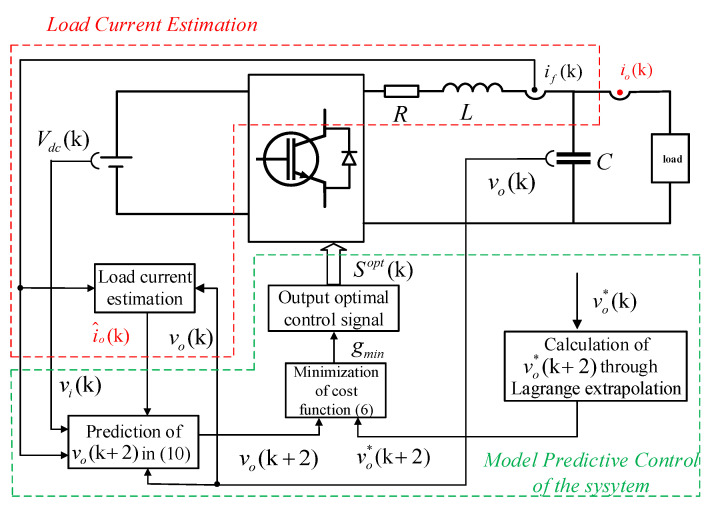
Block diagram of proposed control system.

The purpose of MPC is to output a stable sine wave voltage $v_{o}$, and ensure the quality of the voltage. From (4) and (5), it can be known that $v_{o}\left(k+1\right)$ has no direct mathematical relationship with the switch state. To implement voltage control, a two-step prediction model is employed [26]. Figure 3 shows the block diagram of the control system.

The cost function is defined as

(6) $$g=\left|{v^{*}}_{o}\left(k+2\right)-v_{o}\left(k+2\right)\right|,$$

where $v_{o}\left(k+2\right)$ is the two-step predicted output voltage. ${v_{o}}^{*}\left(k+2\right)$ is the reference voltage derived through fourth-order Lagrange extrapolation. (4) and (5) can be transformed as:

(7) $$i_{f}\left(k+1\right)=\left(\frac{L_{f}-RT_{s}}{L_{f}}\right)i_{f}\left(k\right)+\frac{T_{s}}{L_{f}}\left[v_{i}\left(k\right)-v_{o}\left(k\right)\right],$$

(8) $$v_{o}\left(k+1\right)=v_{o}\left(k\right)+\frac{T_{s}}{C_{f}}\left[i_{f}\left(k\right)-i_{o}\left(k\right)\right].$$

In the two-step horizon time, the two-step predicted output voltage $v_{o}\left(k+2\right)$, is described as below:

(9) $$v_{o}\left(k+2\right)=\left[i_{f}\left(k+1\right)-i_{o}\left(k+1\right)\right]\frac{T_{s}}{C_{f}}+v_{o}\left(k+1\right),$$

Substituting (7) and (8) into (9), it becomes:

(10) $$\begin{array}{cc} v_{o}\left(k+2\right)= & \left(\frac{2}{C_{f}}-\frac{RT_{s}}{C_{f}L_{f}}\right)i_{f}T_{s}\left(k\right)-\frac{T_{s}}{C_{f}}\left[i_{o}\left(k\right)+i_{o}\left(k+1\right)\right]\\ \; & +\left(1-\frac{T_{s}^{2}}{C_{f}L_{f}}\right)v_{o}\left(k\right)+\frac{T_{s}^{2}}{C_{f}L_{f}}v_{i}\left(k\right), \end{array}$$

Therefore, the output voltage depends on the switch state. The value of the load current in the (k+1)th instant, is obtained by Lagrange extrapolation:

(11) $$\begin{array}{c} i_{o}\left(k+1\right)=-i_{o}\left(k-3\right)+4i_{o}\left(k-2\right)-6i_{o}\left(k-1\right)+4i_{o}\left(k\right), \end{array}$$

Meanwhile, the extrapolation of the reference voltage can also be realized by the fourth-order Lagrange extrapolation method as follows:

(12) $$\begin{array}{c} v_{o}^{*}\left(k+2\right)=-4v_{o}^{*}\left(k-3\right)+20v_{o}^{*}\left(k-2\right)-15v_{o}^{*}\left(k-1\right)+10v_{o}^{*}\left(k\right). \end{array}$$

Then, the cost function is calculated for four switching states of the inverter separately. The switching state that has the minimal cost function value is selected to be used in the next time interval.

## 4. Load Current Estimation

To reduce the number of sensors, the sensor that collects the load current signal is removed. Instead, an observer is designed to estimate the periodic load current. Since the significance of reducing the number of sensors has been presented in detail in the Introduction, this section shows how to design the observer, to obtain accurate load current estimation.

### 4.1. Observer Design

The load current is calculated from (5), which is the easiest way to estimate it. However, the derivative term in this equation makes the estimation of the load current susceptible to the interference of high-frequency harmonics, because of the existence of noise or disturbances in the output voltage. To avoid this problem, a harmonic observer is designed in this paper which estimates the load current in the form of finite Fourier series.

Assuming the periodic load current signal to be measured $i_{o}\left(k\right)$ as

(13) $$\begin{array}{cc} i_{o}\left(t\right) & =a_{0}+\sum_{i=1}^{n}a_{i}\cos\left(i\omega t\right)+b_{i}\sin\left(i\omega t\right), \end{array}$$

where $a_{0}$ represents the DC component of $i_{o}\left(t\right)$, $a_{i}\cos\left(i\omega t\right)+b_{i}\sin\left(i\omega t\right)$ denotes the component with frequency iω, ω=2πf is the angular frequency, and f=50 Hz. If $\left\{\left.a_{0},a_{i},b_{i}\right\}\right.\left(i=1,2,\cdots ,n\right)$ can be calculated, the load current can be estimated.

Define $x={\left[x_{1}\;\cdots \;x_{2n+1}\;x_{2n+2}\right]}^{T}={\left[v_{o}\;a_{0}\;a_{1}\;b_{1}\;\cdots \;a_{n}\;b_{n}\right]}^{T}$. In the single-phase inverter model, the differential of the output voltage is

(14) $$\begin{array}{c} {\dot{v}}_{o}\left(t\right)=\frac{i_{f}\left(t\right)-i_{o}\left(t\right)}{C_{f}}. \end{array}$$

Since $a_{i}\cos\left(i\omega t\right)+b_{i}\sin\left(i\omega t\right)$ changes slowly within a sampling period in steady state, its differential is approximately 0. Combining (13) and (14), a dynamic system can be established as

(15) $$\begin{array}{c} \left\{\begin{array}{c} \dot{x}=Ax+Bu,\\ y=Cx, \end{array}\right. \end{array}$$

where

(16) $$A\;=\;-{\left[\begin{array}{cccccc} 0 & \frac{1}{C} & \frac{cos\omega t}{C} & \cdots & \frac{cos(n\omega t)}{C} & \frac{sin(n\omega t)}{C}\\ 0 \end{array}\right]}_{\left(2n+2)\times (2n+2\right)}$$

(17) $$B\;=\;{\left[\begin{array}{cccccc} \frac{1}{C} & 0 & 0 & \cdots & 0 & 0 \end{array}\right]}_{(2n+2)\times 1}^{T}$$

(18) $$u\;=\;i_{f},$$

(19) $$C\;=\;{\left[\begin{array}{cccccc} 1 & 0 & 0 & \cdots & 0 & 0 \end{array}\right]}_{1\times (2n+2)}$$

Based on the state space equation above, a time-varying harmonic observer is established as

(20) $$\begin{array}{c} \left\{\begin{array}{c} \hat{\dot{x}}=A\hat{x}+Bu-l\left(\hat{y}-y\right),\\ \hat{y}=C\hat{x}, \end{array}\right. \end{array}$$

where

(21) $$l=-{\left[-\frac{l_{0}}{C}\;\frac{l_{a_{0}}}{C}\;\frac{l_{a_{1}}cos\left(\omega t\right)}{C}\;\;\cdots \;\frac{l_{a_{n}}\cos\left(n\omega t\right)}{C}\;\frac{l_{b_{n}}\sin\left(n\omega t\right)}{C}\right]}^{T}$$

l is the feedback matrix of the deviation between y and $\hat{y}$. $l_{0},l_{a_{0}},l_{a_{1}},l_{b_{1}},\cdots ,l_{a_{n}},l_{b_{n}}$ are feedback coefficients to be designed.

By defining $l_{0}^{{}^{\prime }}=\frac{l_{0}}{C},l_{a_{i}}^{{}^{\prime }}=-\frac{l_{a_{i}}}{C}$, and $l_{b_{i}}^{{}^{\prime }}=-\frac{l_{b_{i}}}{C}$, and using the Euler formula with the sampling interval $T_{s}$, the discrete-time form of the observer can be written as:

(22) $$\left\{\begin{array}{cc} {\hat{v}}_{o}\left(k+1\right)= & \left\{\frac{i_{f}\left(k\right)-{\hat{i}}_{o}\left(k\right)}{C}-l_{0}^{{}^{\prime }}\left[{\hat{v}}_{o}\left(k\right)-v_{o}\left(k\right)\right]\right\}T_{s}+{\hat{v}}_{o}\left(k\right),\\ {\hat{a}}_{0}\left(k+1\right)= & -T_{s}l_{a_{0}}^{{}^{\prime }}\left[{\hat{v}}_{o}\left(k\right)-v_{o}\left(k\right)\right]+{\hat{a}}_{0}\left(k\right),\\ {\hat{a}}_{i}\left(k+1\right)= & -T_{s}l_{a_{i}}^{{}^{\prime }}\cos\left(i\omega t\right)\left[{\hat{v}}_{o}\left(k\right)-v_{o}\left(k\right)\right]+{\hat{a}}_{i}\left(k\right),\\ {\hat{b}}_{i}\left(k+1\right)= & -T_{s}l_{b_{i}}^{{}^{\prime }}\sin\left(i\omega t\right)\left[{\hat{v}}_{o}\left(k\right)-v_{o}\left(k\right)\right]+{\hat{b}}_{i}\left(k\right),\\ {\hat{i}}_{o}\left(k+1\right)= & {\hat{a}}_{0}\left(k+1\right)+\sum_{i=1}^{n}\left[{\hat{a}}_{i}\left(k+1\right)\cos\left(i\omega t\right)+{\hat{b}}_{i}\left(k+1\right)\sin\left(i\omega t\right)\right]. \end{array}\right.$$

As (22) estimates the value of the load current in *k* + 1, so the Lagrange extrapolation of $i_{o}$ in (11) is needless in the control system when an observer is used.

### 4.2. Proof of Observer Effectiveness

To evaluate the effectiveness of the observer, the dynamic system about the deviation between x and $\hat{x}$ is constructed, by combining (15) and (20). The deviation between x and $\hat{x}$ is set as $\tilde{x}=x-\hat{x}$.

(23) $$\begin{array}{c} \dot{\tilde{x}}=\left(A-lC\right)\tilde{x}, \end{array}\;$$

Let D=A−lC, where

$$\begin{array}{c} D\;=\;\left[\begin{array}{ccccccc} \;-l_{0} & -1 & -\cos\omega t & \cdots & -\cos(n\omega t) & -\sin(n\omega t)\\ \;l_{a_{0}} & 0 & 0 & \cdots & 0 & 0\\ \;l_{a_{1}}\cos\omega t & 0 & 0 & \cdots & 0 & 0\\ \;l_{b_{1}}\sin\omega t & 0 & 0 & \cdots & 0 & 0\\ \vdots & \vdots & \vdots & \vdots & \ddots & \vdots & \vdots \\ \;l_{a_{n}}\cos\left(n\omega t\right) & 0 & 0 & \cdots & 0 & 0\\ \;l_{b_{n}}\sin\left(n\omega t\right) & 0 & 0 & \cdots & 0 & 0 \end{array}\right]\frac{1}{C} \end{array}$$

When t→∞, if $\tilde{x}\to 0$, it means $\hat{x}$ converges to x. Then, $a_{i}\left(i=0,\cdots ,n\right)$ and $b_{i}\left(i=1,\cdots ,n\right)$ can be obtained.

Defining a Lyapunov function as $V\left(\tilde{x}\right)=\frac{1}{2}{\tilde{x}}^{T}P\tilde{x}$, where $P=diag\{1,\frac{1}{l_{a_{0}}},\frac{1}{l_{a_{1}}},\frac{1}{l_{b_{1}}},\cdots ,\frac{1}{l_{a_{n}}},\frac{1}{l_{b_{n}}}\}$. The differentiation of $V(\tilde{x})$ can be written as

(24) $$\begin{array}{cc} \dot{V}\left(\tilde{x}\right) & =\frac{1}{2}\left[{\dot{\tilde{x}}}^{T}P\tilde{x}+{\tilde{x}}^{T}P\dot{\tilde{x}}\right]\\ \; & =\frac{1}{2}\left[{\left(D\tilde{x}\right)}^{T}P\tilde{x}+{\tilde{x}}^{T}P\left(D\tilde{x}\right)\right]\\ \; & ={\tilde{x}}^{T}\mathrm{PD}\tilde{x}\\ \; & =-\frac{l_{0}}{C}{\tilde{x}}_{1}^{2}. \end{array}\;$$

P is a positive matrix when $l_{0},l_{a_{0}},l_{a_{1}},l_{b_{1}},\cdots ,l_{a_{n}}$, and $l_{b_{n}}$ are greater than zero and $V(\tilde{x})$ is a positive function. So that $\dot{V}\left(\tilde{x}\right)⩽0$.

If $\dot{V}\left(\tilde{x}\right)\equiv 0$, then ${\tilde{x}}_{1}\equiv 0$.

(25) $${\tilde{x}}_{2}+{\tilde{x}}_{3}\cos\omega t+\cdots +{\tilde{x}}_{2n+1}\cos\left(n\omega t\right)+{\tilde{x}}_{2n+2}\sin\left(n\omega t\right)\equiv 0.$$

Since {1,cosωt,sinωt,⋯cos(nωt),sin(nωt)} are linearly independent, (25) has the unique solution ${\left[{\tilde{x}}_{2},\cdots {\tilde{x}}_{2n+2}\right]}^{T}=0$. In a word, $\dot{V}\left(\tilde{x}\right)\equiv 0$ only when $\tilde{x}=0$. The dynamic system described in (23) is global asymptotically stable according to the Lyapunov stability theorem, which means that $\tilde{x}$ will converge to 0 when t→∞. The is the proof of the effectiveness of the proposed observer.

The performance of the observer is mainly affected by feedback coefficients, however, there is no theorem on how to choose their optimal values. $l_{0},l_{a_{0}},l_{a_{1}},l_{b_{1}},\cdots ,l_{a_{n}},l_{b_{n}}$ are chosen based on previous experience.

## 5. Simulation and Experimental Results

To examine the performance of the proposed observer-based load current sensorless FCS-MPC scheme, several simulations and experiments are conducted in the studied system, under linear and nonlinear loads. Comparisons with typical estimation methods (low-pass filtering [36] and Kalman filtering [37]) are investigated, to illustrate the superior performance of our scheme. The system parameters of the simulations and experiments are shown in Table 1.

### 5.1. Simulation Results

**Figure 4:**
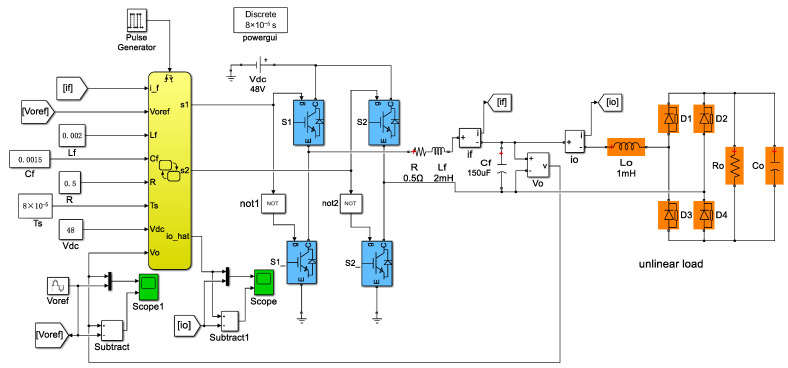
System simulation model.

**Figure 5:**
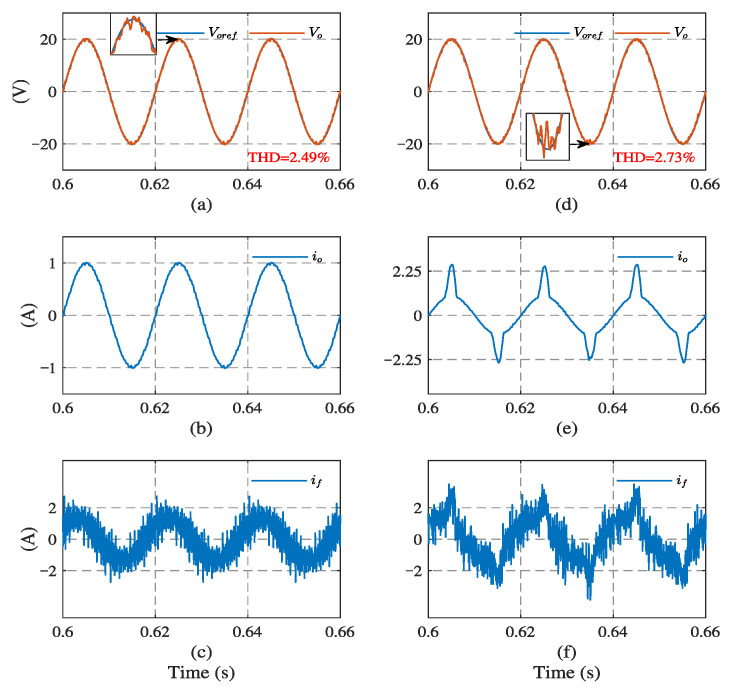
System simulation results with load current sensor. Under linear load: (**a**) output voltage, (**b**) load current, and (**c**) filter current. Under nonlinear load: (**d**) output voltage, (**e**) load current, and (**f**) filter current.

Simulations were carried out using Matlab/Simulink, and the system Simulink Model is shown in Figure 4. The control algorithm was implemented based on Stateflow. Firstly, the two-step model predictive control was implemented with a traditional load current sensor, as comparison to sensorless FCS-MPC strategies. The system performance with load current sensor under different loads is presented in Figure 5. The reference voltage of the output was 20 V, 50 Hz. In the case of linear load, it can be seen that the load current and output voltage are both sinusoidal and their amplitudes are proportional. The LC filter attenuates high-frequency harmonics in the filter current at the output of the inverter. The total harmonic distortion (THD) of the output voltage is 2.49%. Under the nonlinear load, the load current and filter current are distorted and the output voltage of the THD is 2.73%.

**Figure 6:**
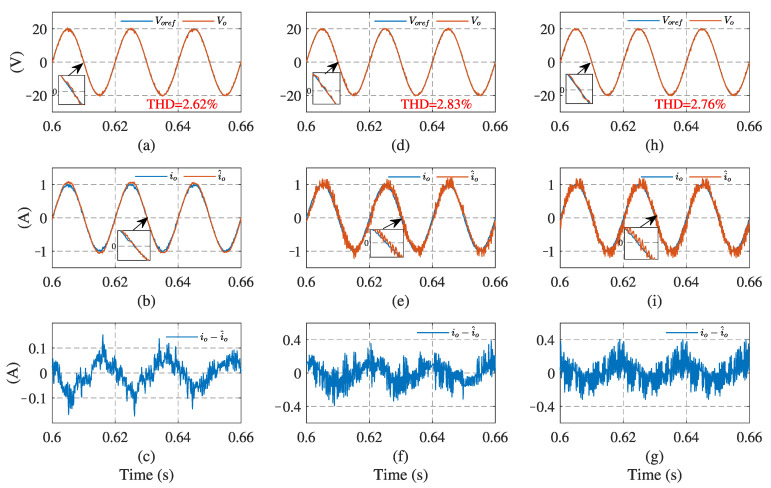
System simulation results using the current estimation method under linear load: (**a**,**d**,**h**) output voltage of observer, Kalman filter, and low-pass filter, respectively; (**b**,**e**,**i**) load currents of the three methods, respectively; (**c**,**f**,**g**) current estimation errors of the three methods, respectively.

**Figure 7:**
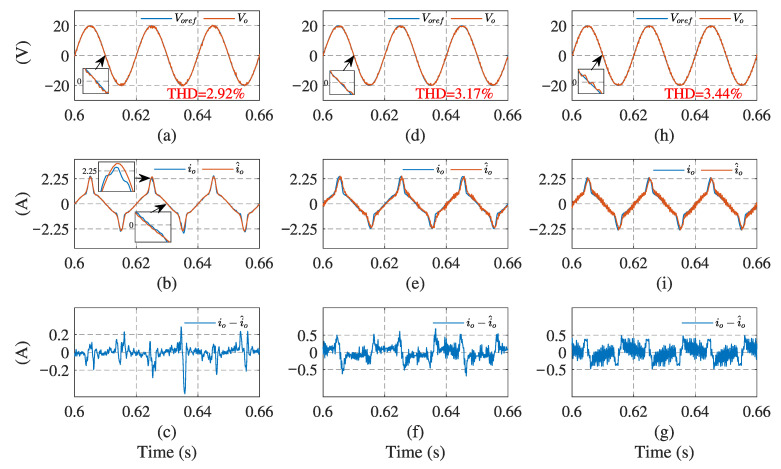
System simulation results using current estimation method under nonlinear load: (**a**,**d**,**h**) output voltage of observer, Kalman filter, and low-pass filter, respectively; (**b**,**e**,**i**) load currents of the three methods, respectively; (**c**,**f**,**g**) current estimation errors of the three methods, respectively.

Figure 6 and Figure 7 illustrate the simulation results with the proposed observer-based FCS-MPC, Kalman filter, and low-pass filter sensorless control methods, under linear and nonlinear loads, respectively. The output voltage reference $v_{oref}$, output voltage $v_{o}$, load current io, load current estimation ${\hat{i}}_{o}$, and estimation error are presented. The results show that two filtering methods provide a similar performance under linear loads, while Kalman filtering performs better with a nonlinear load. Compared with the filtering methods, the proposed observer has the best performance in different load conditions.

In addition, the root mean square error (RMSE) [27] is introduced, to quantify the current estimation performance. The quantitative simulation comparisons of the load current RMSE and output voltage THD under different loads, are presented in Table 2. The RMSE of the proposed observer is much lower than the other two filtering methods. In terms of output voltage performance, the THD deviations between the proposed control scheme and typical sensor-based method, under linear and nonlinear loads, are less than 0.2%. The proposed method has the lowest THD value among the three estimation methods. It can be concluded that the proposed sensorless control scheme has a better estimation accuracy and output voltage tracking performance than the other methods, under both linear and nonlinear loads.

### 5.2. Experimental Results

**Figure 8:**
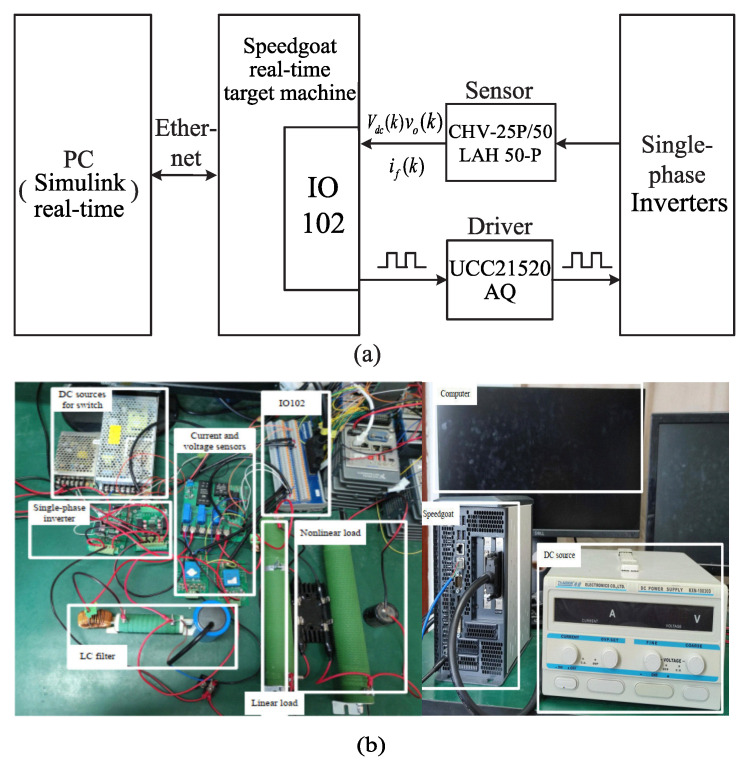
Experimental test bench: (**a**) block diagram, (**b**) experimental platform.

Figure 8 shows the composition of the rapid control prototype in detail. Figure 8a is the block diagram illustrating the work principle of the test bench, and Figure 8b shows the structure of the experimental platform. The power stage of the UPS inverters are constructed with silicon carbide power MOSFET C2M0080120. Based on the Simulink Real-Time toolbox of MathWorks, the proposed control algorithm was implemented in the Speedgoat real-time target, which was used as the controller of the rapid control prototype (RCP). IO102 is an I/O module as the interface between the controller and single-phase inverters. In addition, Zhaoxin KXN-10030D was used as the DC power supply. The current signals were collected by the LAH 50P sensor and the voltage signals were collected by the CHV-25P/50 sensor.

**Figure 9:**
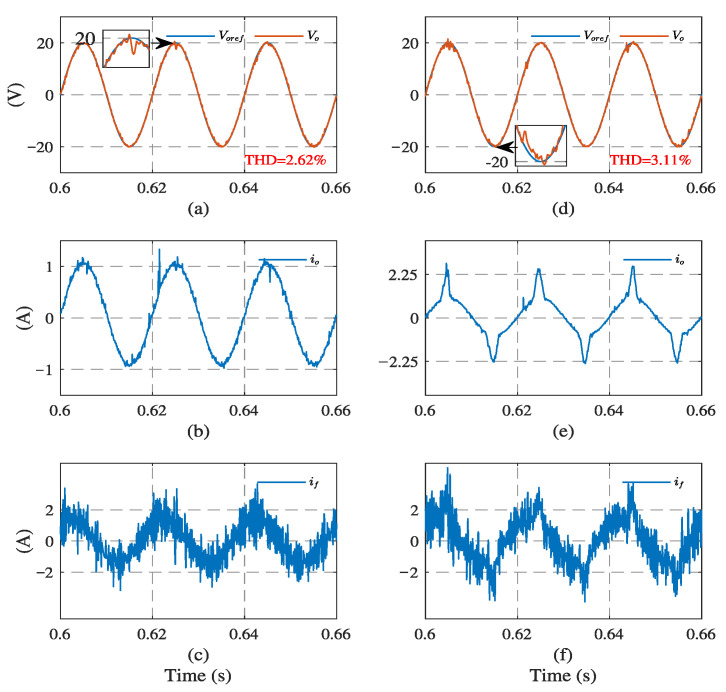
System experimental results with load current sensor. Under linear load: (**a**) output voltage, (**b**) load current, and (**c**) filter current. Under nonlinear load: (**d**) output voltage, (**e**) load current, and (**f**) filter current.

The experimental results of the system using the load current sensor are shown in Figure 9. Due to the system noise and sensor error existing in the experiment, some peaks and fluctuations occasionally occur in the waveforms of the load current. Therefore, the output voltage THD is 2.62% under a linear load and 3.11% under a nonlinear load, slightly larger than the simulation results.

**Figure 10:**
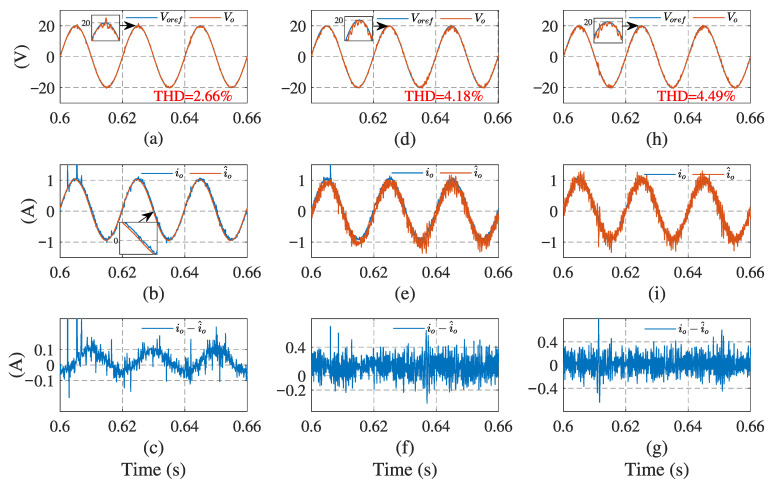
System experimental results using current estimation method under linear load: (**a**,**d**,**h**) output voltage of observer, Kalman filter, and low-pass filter, respectively; (**b**,**e**,**i**) load currents of the three methods, respectively; (**c**,**f**,**g**) current estimation errors of the three methods, respectively.

**Figure 11:**
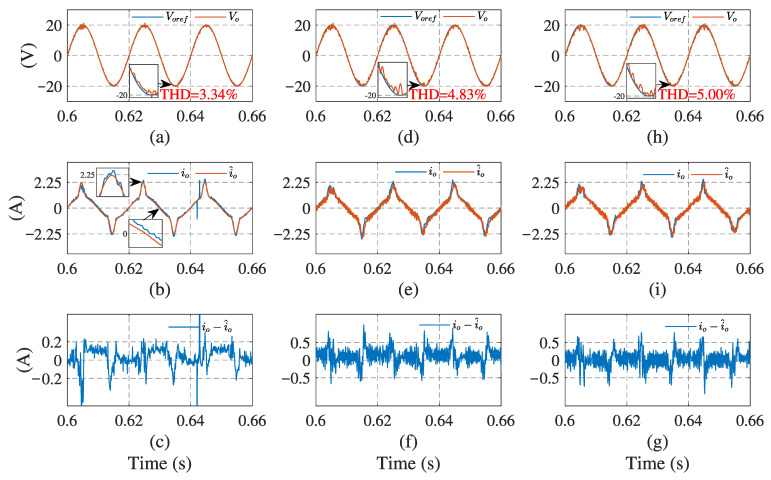
System experimental results using current estimation method under nonlinear load: (**a**,**d**,**h**) output voltage of observer, Kalman filter, and low-pass filter, respectively; (**b**,**e**,**i**) load currents of the three methods, respectively; (**c**,**f**,**g**) current estimation errors of the three methods, respectively.

Figure 10 and Figure 11 present the control system experimental results using the proposed observer-based FCS-MPC and two filtering sensorless methods, under the linear and nonlinear loads. As we can see, using the observer, the current estimation error is less and the output voltage follows its reference voltage waveform better than with the other two sensorless methods. The quantitative comparisons of the load current RMSE and output voltage THD under different loads, are presented in Table 3. It can be seen that the load current RMSE value of the proposed observer method is 0.0859 A and its voltage THD is 2.66% under linear load and 3.34% under nonlinear load, which are all the lowest among the three estimation methods. It can be concluded that the proposed sensorless control scheme has a better estimation performance and lower output voltage THD. The experimental results confirm the conclusion obtained from the simulation.

**Figure 12:**
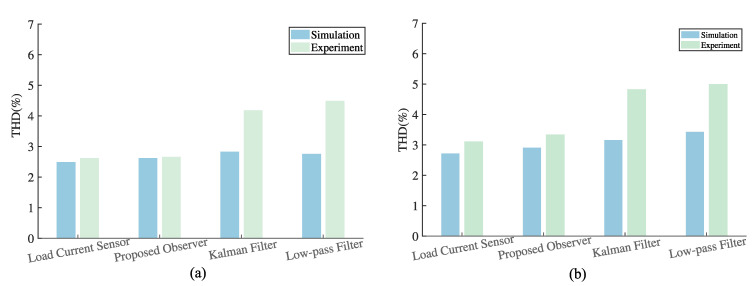
Comparison of simulation and experimental output voltage THD values: (**a**) linear load, (**b**) nonlinear load.

Furthermore, Figure 12 shows the comparison of the simulated and experimental output voltage THD values, in the form of histograms. Under the same control scheme, the THD of the experiment is a little higher than that of the simulation, due to noise. From the histogram, it can be clearly seen that the output voltage THD of the proposed observer method, is very close to that of the traditional method with a load current sensor in both simulations and experiments. Compared with the other two sensorless methods, the THD of our method is much lower in the experiment, which illustrates the effectiveness of the proposed control scheme.

**Figure 13:**
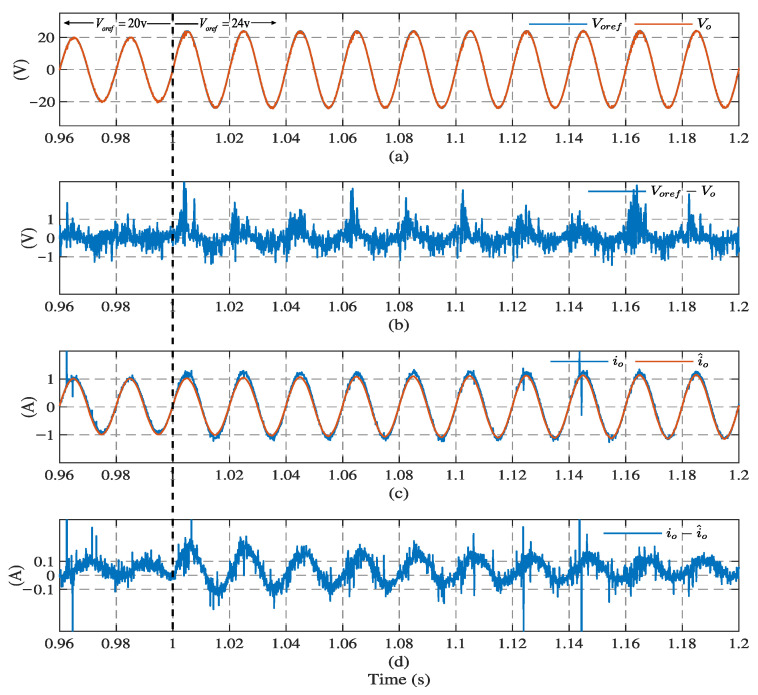
System dynamic response results using observer under linear load: (**a**) output voltage, (**b**) voltage tracking error, (**c**) load current, and (**d**) current estimation error.

**Figure 14:**
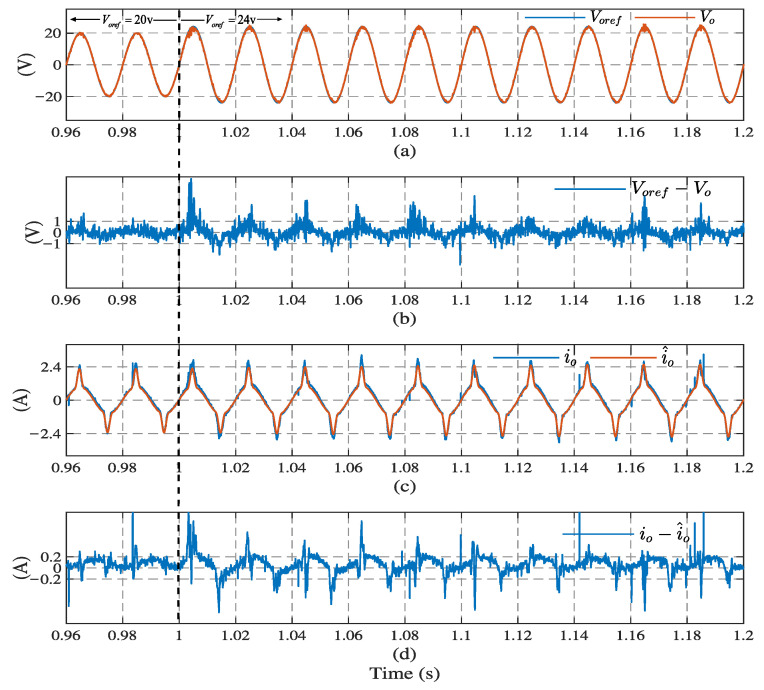
System dynamic response results using observer under nonlinear load: (**a**) output voltage, (**b**) voltage tracking error, (**c**) load current, and (**d**) current estimation error.

Figure 13 and Figure 14, show the transient response using the proposed observer-based FCS-MPC scheme when the reference voltage amplitude changes from 20 V to 24 V at 1 s, with linear and nonlinear loads, respectively. It can seen that the output voltage quickly recovers to the new reference voltage waveform after a short transient, under the two kinds of load (0.02 s under the linear load and 0.1 s under the nonlinear load). It takes the observer estimation current 0.04 s under the linear load and 0.14 s under the nonlinear load, to converge to the load current. It should be noted that, as the observer estimates the load current through the output voltage, the current estimation response time will be longer than the voltage tracking response time.

## 6. Conclusions

In this paper, an observer-based load current estimation FCS-MPC algorithm for single-phase UPS inverters is proposed. The load current RMSE value of the proposed observer method is 0.0859 A, the output voltage THD is 2.66% under linear load and 3.34% under nonlinear load, in the experiment. The difference of THD values between the proposed control scheme and traditional sensor-based methods is less than 0.25%. Our load current RMSE and output voltage THD values are all the lowest among the three estimation methods. Assessed by the necessary simulations and experiments, the observer-based sensorless method has a better performance in load current estimation and output voltage tracking under different load conditions, compared with the typical Kalman filter and low-pass filter sensorless methods. Moreover, as the estimated load current is composed of finite harmonics, the observer can achieve noise elimination, leading to a higher level of reliability in the control system.

## Figures and Tables

**Table 1:** System electrical parameters and control parameters.

Parameter	Value
Input DC voltage ($V_{dc}$)	48 V
Filter inductance ($L_{f}$)	2 mH
Filter capacitance ($C_{f}$)	150 μF
System resistance (*R*)	0.5 Ω
Linear load resistance ($R_{L}$)	20 Ω
Load inductance ($L_{o}$)	1 mH
Load resistance ($R_{o}$)	80 Ω
Load capacitance ($C_{o}$)	470 μF
Sampling interval ($T_{s}$)	80 μs

**Table 2:** Simulation result comparisons of the load current RMSE and output voltage THD, under different loads.

Load Type	With Load Current Sensor		Proposed Observer		Kalman Filter		Low-Pass Filter	
	RMSE (A)	THD (%)	RMSE (A)	THD (%)	RMSE (A)	THD (%)	RMSE (A)	THD (%)
Linear Load	-	2.49	0.0531	2.62	0.1817	2.83	0.1341	2.76
Nonlinear Load	-	2.73	0.0798	2.92	0.2156	3.17	0.2222	3.44

**Table 3:** Experimental result comparisons of the load current RMSE and output voltage THD under different loads.

Load Type	With Load Current Sensor		Proposed Observer		Kalman Filter		Low-Pass Filter	
	RMSE (A)	THD (%)	RMSE (A)	THD (%)	RMSE (A)	THD (%)	RMSE (A)	THD (%)
Linear Load	-	2.62	0.0859	2.66	0.2027	4.18	0.1809	4.49
Nonlinear Load	-	3.11	0.1506	3.34	0.2704	4.83	0.2533	5.00

## Data Availability

Not applicable.

## Nomenclature

$V_{dc}$	Input DC voltage
$v_{i}$	Output voltage of H-bridge
$v_{o}$	Inverter output voltage
$v_{o}^{*}$	Inverter output voltage reference
$i_{f}$	Filter input current
$i_{o}$	Load current
${\hat{i}}_{o}$	Estimation load current
*g*	Cost function
$C_{f}$	Filter capacitor
$L_{f}$	Filter inductance
*R*	System resistance
$R_{L}$	Linear load resistance
$L_{o}$	Load inductance
$R_{o}$	Load resistance
$C_{o}$	Load capacitance
$T_{s}$	Sampling interval
$a_{0}$	DC component of load current
$a_{i},b_{i}$	Harmonic coefficient of load current
$l_{i}$	Feedback coefficient
